# Interaction and Compatibility Studies in the Development of Olmesartan Medoxomil and Hydrochlorothiazide Formulations under a Real Manufacturing Process

**DOI:** 10.3390/pharmaceutics14020424

**Published:** 2022-02-16

**Authors:** Mac Arturo Murillo-Fernández, Ernesto Montero-Zeledón, Ariadna Abdala-Saiz, José Roberto Vega-Baudrit, Andrea Mariela Araya-Sibaja

**Affiliations:** 1 Escuela de Física, Instituto Tecnológico de Costa Rica, Cartago 159-7050, Costa Rica; mamurillo@itcr.ac.cr (M.A.M.-F.); emontero@itcr.ac.cr (E.M.-Z.); 2 Departamento de Investigación y Desarrollo, Calox de Costa Rica, San José 126224-01, Costa Rica; aabdala@calox.com; 3 Laboratorio Nacional de Nanotecnología LANOTEC-CeNAT-CONARE, Pavas, San José 1174-1200, Costa Rica; jvegab@gmail.com; 4 Laboratorio de Investigación y Tecnología de Polímeros POLIUNA, Escuela de Química, Universidad Nacional de Costa Rica, Heredia 86-3000, Costa Rica

**Keywords:** olmesartan medoxomil, hydrochlorothiazide, antihypertensive, solid–state characterization, compatibility study, excipients, eutectic mixtures

## Abstract

A drug–drug and drug–excipient interactions and compatibilities study was conducted for two fixed-dose combination (FDC) products containing olmesartan medoxomil (OLM)/hydrochlorothiazide (HCT) 20/12.5 mg and OLM/HCT 40/12.5 mg during their development including storage. The study consisted of the evaluation of samples retrieved during all stages of a real manufacturing process. Powder X-ray diffraction (PXRD), differential scanning calorimetry (DSC), thermogravimetry (TGA), Fourier transform infrared spectroscopy (FT-IR), and contact angle techniques were applied to the samples to determine interactions and incompatibilities. Dissolution tests and long-term stability studies were conducted to evaluate dosage form performance. Results showed weak solid–state interactions able to obtain a eutectic mixture of OLM and HCT while microcrystalline cellulose (MC) impacted the thermal stability of both drugs. Reliable dissolution and long-term stability tests confirmed that the interactions observed were not considered incompatibilities because they were not influenced by the performance of the final products.

## 1. Introduction

Hypertension or raised blood pressure (BP), is a serious medical condition that contributes to the development of cardiovascular diseases [1]. According to the World Health Organization (WHO) over a billion people have the condition and it represents a major cause of premature death worldwide [2]. Further, it has been reported by the WHO that only one in five people with hypertension have the problem under control where two-thirds of cases are found in low- and middle-income countries. In this regard, patients with Stage 2 hypertension (systolic BP ≥ 160 mmHg or diastolic ≥ 100 mmHg), have achieved adequate control of BP only by drug combination therapy. Consequently, the combination of antihypertensive agents with complementary mechanisms of action [3,4,5,6] is recommended. Studies have reported that the addition of hydrochlorothiazide (HCT) improves the BP lowering effects of angiotensin II receptor blockers (ARBs) and, angiotensin-converting enzyme (ACE) inhibitors. This enhancement has been attributed to the activation of the renin–angiotensin system (RAS) by the HCT, turning BP more dependent on angiotensin II [7].

Several fixed-dose combinations (FDCs) containing HCT with β-blockers, ACE inhibitors, ARB along with ACE inhibitors, and calcium channel blockers are now available. However, monotherapy [8,9] compared to other ACE inhibitors [10,11] or to different antihypertensive drug classes [12,13], the one containing olmesartan medoxomil (OLM) and HCT has been demonstrated in clinical trials to be the most effective in significant BP reductions and BP control in many patients while it is a well-tolerated therapy [3,14], hence, increasing the interest in developing formulations containing the OLM-HCT combination.

In this context, the development of a pharmaceutical dosage form is essential to conduct compatibility studies between the drug and excipients as well as between the drugs in the case of FDC formulations. Compatibility studies intend to guarantee the safety, efficacy, and stability of the final product by detecting chemical and/or physical instability [15] during the manufacturing, packaging, and storage processes [16]. The drug instability in the dosage form is generally mediated by solid–state reactions as complexation, ion exchange, polymorphic transformation, the formation of eutectic or solid solutions among others [16,17,18].

**Figure 1:**
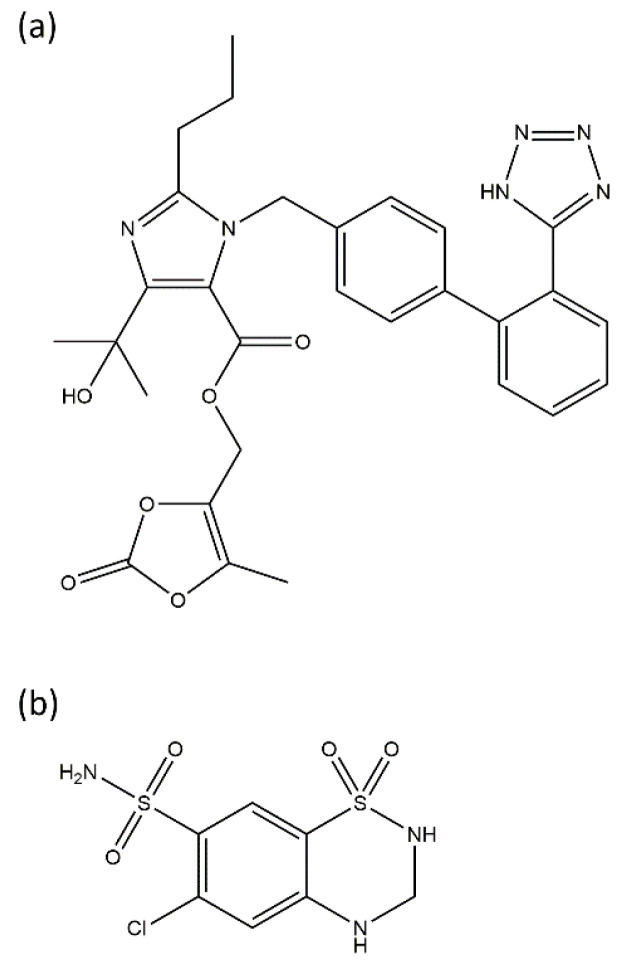
Chemical structures of (**a**) olmesartan medoxomil (OLM) and, (**b**) hydrochlorothiazide (HCT).

Compatibility studies are commonly carried out evaluating 1:1 drug–drug, and drug–excipients mixture combinations prepared in laboratory conditions [19]. Therefore, in this contribution, a compatibility study between excipients and OLM and HCT as well as between the drugs in real manufacturing conditions is reported. In addition, a long-term stability test has been conducted at the final stage of launching the product to the market. Figure 1 shows the chemical structures of drugs involved in this study.

## 2. Materials and Methods

### 2.1. Materials 

OLM and HCT raw materials both in their polymorphic form I and microcrystalline cellulose (MC) excipients (Avicel^®^ 101 and Vivapur^®^), croscarmellose sodium (Vivasol^®^), mannitol 2080 (Mannogem^®^), magnesium stearate, corn starch (Starch 1500), lactose (Lactose Fast-Flo), and colloidal silicon dioxide (Aerosil^®^ 200) were supplied by Calox de Costa Rica (San José, Costa Rica). All excipients and drugs were National Formulary, USP grade. Olmesartan medoxomil and hydrochlorothiazide reference standards used in the HPLC quantification method were purchased from USP.

### 2.2. Methods

#### 2.2.1. Sample Preparation

**Figure 2:**
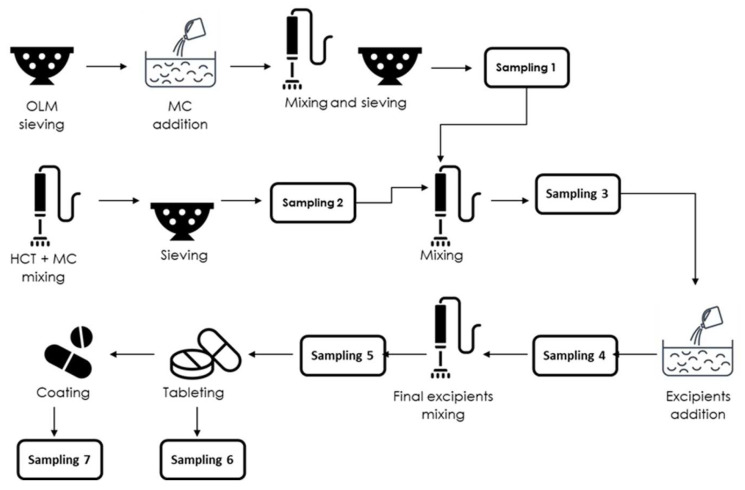
Schematic representation of the manufacturing process.

In this study, compatibilities and interactions between drugs and excipients were evaluated for two formulations of OLM–HCT tablets. The first one is composed of OLM 40 mg and HCT 12.5 mg (F_1_) and, the second one contains OLM 20 mg and HCT 12.5 mg (F_2_). Drug–drug and drug–excipient mixtures were prepared using appropriate quantities of all components necessary to obtain 28 kg of each formulation. The solids were prepared accordingly to the schematic process presented in Figure 2. and, submitted to real manufacture conditions, which include tableting and coating processes. The specific conditions are reserved to maintain the confidentiality of the pharmaceutical industry involved in this study.

#### 2.2.2. Powder X-ray Diffraction (PXRD)

PXRD patterns were recorded on a PANalytical Empyrean diffractometer (Panalytical, Almelo, The Netherlands), equipped with a PIXcel detector (Medipix2, CERN, Geneva, Switzerland). Samples were scanned with a Cu Kα source (λ = 1.5418 Å), operated at 45 kV and 40 mA, step size 0.0016°, step time 20 s, and 2θ angular range between 4° and 50°. A soller of 0.04 rad located at the X-ray tube and a large soller of 0.04 rad located at the detector were used. A divergence slit of 1/4° and an antiscatter slit of 1/2° were implemented. Kβ was filtered using nickel. Powder material was measured in a zero-background sample holder whereas tablets were sanded to obtain a flat surface and placed in an irregular shape sample holder for the front-loading of solid samples. All the PXRD data were obtained at 25 °C, environmental conditions. The software Data Collector, High Score plus and PDF4+ (2021, Malvern PANalytical, Malvern, UK) were utilized.

#### 2.2.3. Fourier Transform Infrared Spectroscopy (FT-IR)

FT-IR spectra of pure HCT, OLM, excipients and their mixtures were analyzed using a Thermo Scientific Nicolet 6700 FT-IR spectroscope (Thermo Scientific, Waltham, MA, USA) fitted with a diamond attenuated total reflectance (ATR) accessory. A small amount of the solid was placed directly into the ATR cell without further preparation. The data were recorded in the range of 4000−500 cm^−1^, collecting 32 scans at a resolution of 4 cm^−1^ with an additional correction at 25 cm^−1^ using the OMNIC Software.

#### 2.2.4. Thermal Analysis

Differential scanning calorimetry (DSC) curves of the pure drugs and excipients as well as their mixtures were obtained using a TA Instruments DSC-Q200 calorimeter (New Castle, PA, USA) equipped with a TA Refrigerated Cooling System 90 (New Castle, PA, USA). Approximately 2 mg of the sample was weighted into aluminum crucibles and fitted in the DSC cell. The measurements were conducted under a dynamic nitrogen atmosphere of 50 mL/min, a heating rate of 10 °C/min and a temperature range of 40 to 200 °C or 300 °C when necessary. The DSC cell was calibrated with a standard reference material of indium.

Thermogravimetric analysis (TGA) of the pure drugs, a mixture of them with MC and the sampling five, were performed on a TA Instruments model Q500 thermogravimetric analyzer. Approximately 5 mg of the sample was in place in a platinum crucible using a temperature ramp of 10 °C/min from 25 to 1000 °C under a nitrogen atmosphere flow of 10 mL/min on the sample and 90 mL/min on the microbalance.

#### 2.2.5. Contact Angle Measurement

The contact angle measurement was performed by the sessile drop technique using a Ramé-hart 250 F_1_ goniometer system (Rame-hart, Succasunna, NJ, USA) the capture of the image and the determination of the contact angle were carried out using the Drop-Image software.

#### 2.2.6. Dissolution Test of HCT-OLM Tablets

The dissolution test of both F_1_ and F_2_ tablets were evaluated on a Hanson Elite Vision G2 dissolution test (Hanson Research, Chatsworth, CA, USA) system using the USP dissolution apparatus 2, in 900 mL of HCl 0.1 N as the medium was kept at 37 °C, with a stirring rate of 75 rpm for 30 min. Then, the solutions were filtered using a 0.45 µm membrane filters and analyzed by HPLC to determine drug concentration. The OLM and HCT content were quantified using a previously developed and validated in-house HPLC method (not reported) consisting of a Shimadzu LC-2010A HT HPLC (Shimadzu, Tokyo, Japan) system, equipped with variable wavelength detector, pump, variable temperature compartment column and autosampler. The mobile phase was composed of ammonium phosphate adjusted to pH 3.0 with triethylamine (A) and acetonitrile (B) using a gradient elution method starting at A:B 74:26 to reach 60:40 in 13 min, a flow rate of 1.3 mL/min, 30 µL injection volume, detection at 230 nm in a Phenyl-Hexyl column (Agilent, Santa Clara, CA, USA) (250 mm × 4.6 mm, 5 µm), and a temperature of 30 °C.

#### 2.2.7. Solid-State and Physical Stability Study

A long term stability study was conducted on the final packaged products for the two developed formulations, F_1_ and F_2,_ according to the ICH-Q1F Guideline [20]. The samples used were retention samples from the quality assurance process which were stored in a specific room at natural ambient conditions (i.e., approximately at 30 °C and 75% relative humidity) for 24 months. Then, samples were analyzed by PXRD, DSC, FT-IR, and dissolution test using the same conditions described in the previous sections. In addition, changes in the coating’s color in the product during time storage were determined [21].

Color were measured according to ISO/CIE 11664-2,3,4 standard [22] using a DigiEye^®^ imaging system of VeriVide^®^ (VeriVide, Leicester, UK), equipped with two light sources simulating the CIE D65 illuminant, and a special chamber providing a diffuse and very uniform illumination as well as an RGB digital camera (Nikon D7000^®^, Tokyo, Japan) placed in the vertical to the sample tray. The calibration of the camera was performed by using a ninth-order polynomial fit from RGB measurements of the 273 patches in a standard color chart and by keeping the average color difference between the nominal and measured values of the 273 color patches lower than 1.0 CIELAB unit. Twenty tablets were placed in a black sample holder in order to digitally determine the CIELAB coordinates of both sides of each new and old (24 months) analyzed tablets, to evaluate its color variability and change. To quantify the color variability, the MCDM (Mean Color Difference to the Mean) formula was used; this formula is a standard deviation alternative calculation that assumes the color space perceptual uniformity [23]. To evaluate the color changes, the CIEDE2000 (∆E00) Color Difference formula was used, in accordance with the ISO/CIE 11664-6 standard [24].

## 3. Results

### 3.1. Powder X-ray Powder Diffraction (PXRD)

It is well known that industrial manufacturing processes like drying, milling, micronization, wet granulation, lyophilization, and compaction can cause polymorphic transformations and the unexpected appearance of new crystalline phases [25,26]. Those procedures have been intentionally applied to achieve a physicochemical property modification based on the crystal engineering approach in the light of all possible manipulations of altering crystal packing, to disrupt crystal lattices, and/or reduce the size of the crystal [27]. A search in the literature as well as in the Cambridge Structural Database (CSD) was performed for both drugs. There was no structure deposited for OLM in the CSD and only a few reports on OLM polymorphism in the literature were found [28,29]. After comparison of peaks reported by Qui et al. (2017) [29] and with the peaks observed in the experimental powder pattern, it was concluded that the Form I is the polymorph present in the OLM starting material. In the case of HCT, several deposited structures were found in the CSD of which the calculated patterns were compared to the experimental pattern of the HCT raw material used herein. The experimental pattern matched with Form I of the deposition number 962,494 structure (CSD Entry: HCSBTZ05) [30].

**Figure 3:**
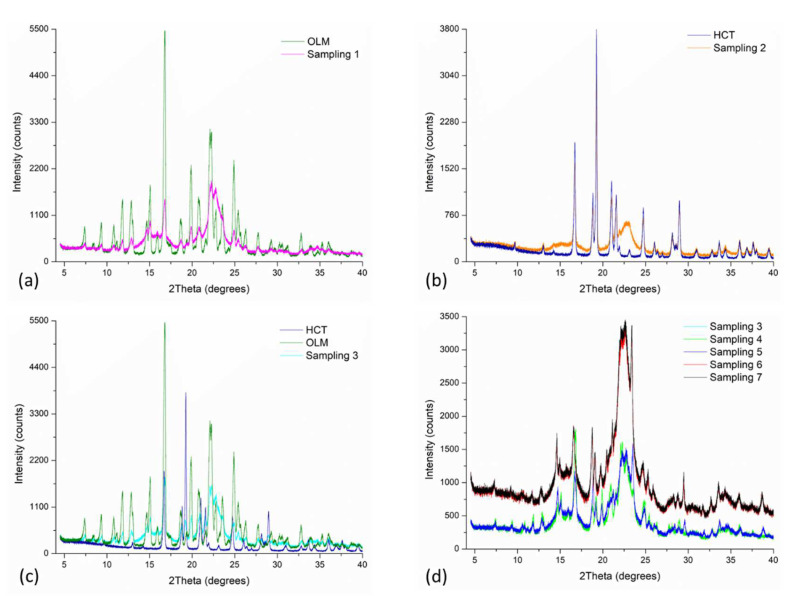
Experimental PXRD patterns of each sampling of F_1_: (**a**) OLM and sampling 1 (i.e., OLM + MC), (**b**) HCT and sampling 2 (i.e., HCT + MC), (**c**) OLM, HCT and sampling 3 (i.e., sampling 1 + 2), (**d**) Sampling 3 and samplings from 4–7.

Figure 3 shows the PXRD patterns of sampling 1 to 5 (see Figure 2) of F_1_ compared with pure drugs’ powder patterns. Only a few and weak reflections were observed in the diffractograms of all the samples which correspond to their respective pure drugs’ powder patterns signals. Therefore, no phase transformation in a strict sense from one to another crystal structure was evidenced; however, the amorphous phase in all the samples predominated. The increase in the amorphous content was observed along with the manufacturing process being more significant in the final steps and products. The amorphization exhibited in the samples was probably provoked by the characteristics of the mixing and sieving process [31,32]. Similar results were observed for the samples withdrawn in the manufacturing of F_2_ presented in Figure S1 (see Supplementary Materials).

### 3.2. Fourier Transform Infrared Spectroscopy (FT-IR)

Intermolecular interactions as hydrogen bonds can occurred between O–H or N–H containing molecules and the O– or N–atom of another molecule. In this case, both drugs possess hydrogen bond donor and acceptor groups [33]. In the case of hydrogen bond formation the wavenumber related to X–H bending is observed at a higher position and the one corresponding with the carbonyl C=O stretching is observed at a lower one [34].

**Figure 4:**
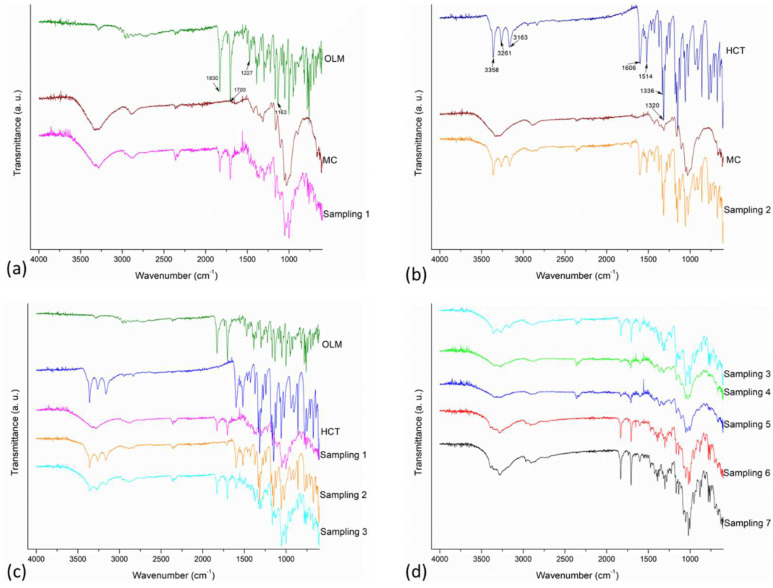
FT-IR spectra of each sampling of F_1_: (**a**) OLM and sampling 1 (i.e., OLM + MC), (**b**) HCT and sampling 2 (i.e., HCT + MC), (**c**) OLM, HCT, and sampling 3 (i.e., sampling 1 + 2), (**d**) Sampling 3 and samplings from 4 to 7.

The FT-IR spectra of each sampling of F_1_ as well as the pure OLM, HCT, and MC are presented in Figure 4. Pure OLM presents two characteristic bands at 1832 cm^−1^ and 1700 cm^−1^ corresponding with the carbonyl group in its lactone and ester nature, respectively, as well as the C–O stretching at 1227 cm^−1^ for the lactone and at 1163 cm^−1^ for the ester group. The NH out-of-plane bending mode of the CNH moiety was observed from 1200 cm^−1^ to 600 cm^−1^, including the peak at 768 cm^−1^, and the NNN bending was observed at 1002 cm^−1^ [35,36]. On the other hand, pure HCT exhibits characteristic absorption bands at 3358 and 3261 cm^−1^ corresponding with the N–H stretching of aliphatic primary and secondary amine. Further, bands associated with the C=C stretching vibrations were observed at 1606 and 1514 cm^−1^; C–H bending of aromatic compounds were evident at 1599 cm^−1^, 1516 cm^−1^ as well as C–H stretching vibrations of aromatic compounds at 3163 cm^−1^. Finally, the absorption bands attributed to SO_2_ were observed at 1336 cm^−1^ and 1320 cm^−1^ for the asymmetric and at 1169 cm^−1^ and 1062 cm^−1^ for the symmetric stretching. [35,36]. MC shows the characteristic cellulose backbone composed by O–H stretching absorption around 3350 cm^−1^, C–H stretching absorption at 2893 cm^−1^ as well as C–O–C stretching absorptions between 900 cm^−1^ and 1200 cm^−1^ [36,37,38]. The FT-IR spectra for all the samplings including tableting and coating show absorption bands in the same position of their individual components indicating no strong interactions in the solid state. Similar results were observed for the samples withdrawn in the preparation of F_2_ (see Figure S2).

### 3.3. Thermal Analysis (TA)

In the study of polymorphic transformations, TA is an important complementary technique and has been the most widely used DSC and TGA [39,40,41].

**Figure 5:**
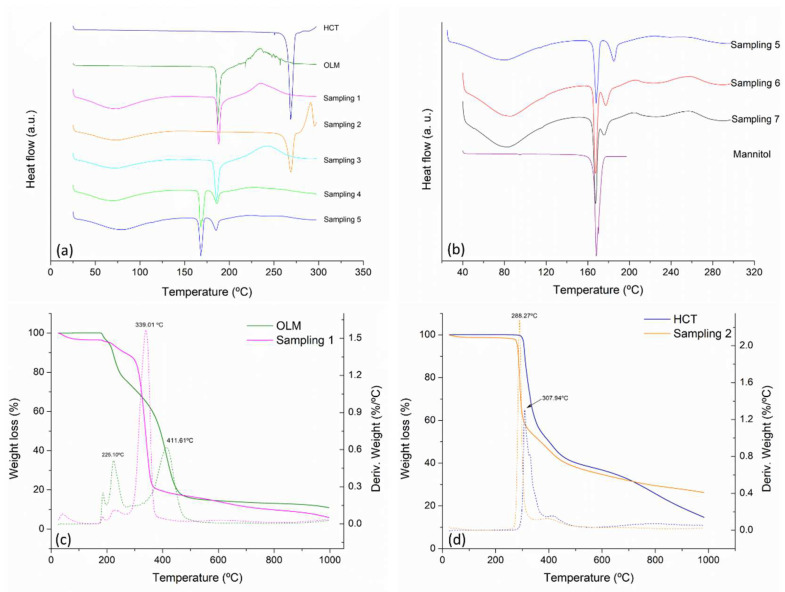
DSC curves of F_1_ (**a**) pure OLM, HCT, and sampling from 1 to 5, (**b**) sampling from 5 to 7 and mannitol, (**c**) thermograms of OLM and sampling 1 and (**d**) thermograms of HCT and sampling 2. (Dotted lines represent the derivative of % weight loss as a function of temperature).

Figure 5a presents the DSC curves obtained for the studied samples as well as for the HCT and OLM raw materials. OLM and HCT melting temperatures were observed at 186.5 °C and 269.1 °C, respectively, which correspond to the reported Form I of them in the literature [28,30]. The DSC curves of sampling 1 and 2 exhibited the thermal endothermic events associated with OLM and HCT without shift. Interestingly, in the case of OLM, the noisy signal observed from 209 °C to 263 °C, which is indicative of thermal degradation was observed as smoothed in sampling 1 which contains MC. On the other hand, the exothermic event at 290.9 °C observed in sampling 2 (e.g., HCT and MC) suggests thermal decomposition at a higher temperature than the pure drug. Therefore, TGA measurements were conducted and presented later herein.

In the sample containing HCT–OLM–MC, a single endothermic event was observed at a lower temperature than of the individual pure drugs, being much lower than the HCT thermal event and slightly lower than the OLM melting temperature. This thermal behavior is commonly observed in eutectic mixture formation [42]. Furthermore, the unaltered crystal structure observed in this sample by PXRD results, as well as DSC results, confirm the formation of a eutectic mixture between HCT and the OLM exhibiting a melting temperature at 185.3 °C. This thermal behavior was observed in the samples withdrawn along the process from sampling 3 to the final product.

Eutectic mixtures is a type of solid dispersion [43] exhibiting differences in properties like solubility, stability, and bioavailability due to their high free energy, enthalpy, and entropy functions [44,45,46]. These characteristics can be advantageous in pharmaceutical formulations to ameliorate or overcome some drawbacks, for instance manufacturing of poorly water-soluble drugs. In addition, preparing them is considered low cost, easier to produce, and scale-up [47,48] and, in terms of regulations, they are not considered new chemical entities [48] or new crystal forms [49].

After the addition of the other excipients in sampling 4, a second melting event at 167.9 °C was observed in the DSC curves as well as in sampling 5, the tableted and coated products. The DSC analyses of the excipients added to the process confirm that this endothermic event corresponds to the melting temperature of mannitol while maintaining the constant melting event of the eutectic mixture formed between OLM and HCT. Hence, no additional drug–excipient interactions have occurred. However, tableting and coating samples presented a decrease in the melting temperature of the eutectic of approximately 10 °C, as evidenced in Figure 5b. Theoretically, the decrease in melting temperature has been attributed to the high pressures applied to particles in a powder [50] during compression or tableting. Bi et al. (2003) have reported the eutectic formation upon compaction [51]. The authors’ hypothesis is that mechanical stress promotes intimate contact between the eutectic’s components replacing the effect of temperature typically needed for eutectic formation, which the only function is indeed to facilitate that intimate contact [51]. The same thermal behavior was presented by the F_2_ formulation (see Figure S3).

The thermal stability of OLM and HCT containing MC was studied comparing the weight loss of the pure drugs and their respective mixtures containing MC through the TGA curves. Figure 5c shows that the first mass loss event of pure OLM occurs at 225.10 °C, while in their mixture with MC, the event was moved to 339.01 °C, an increment of more than 100 °C. As previously suggested by the DSC curves, thermograms of pure HCT in Figure 5d exhibit a major decomposition event at 307.94 °C, whilst sampling 2 decreased to 288.27 °C. These results hint that the MC interacts positively with the OLM, ameliorating its thermal stability. Nevertheless, the MC apparently caused the opposite effect on HCT to a smaller degree because the difference in temperature was around 20 °C.

### 3.4. Contact Angle

Contact angle is related to the degree of wettability or the interaction of a liquid with a solid surface. Usually, the contact angle ranges from 0° to 180° for complete wetting to no wetting, respectively [52]. Table 1 shows the contact angle values for OLM, HCT, and their binary mixtures in the composition of F_1_ and F_2_. The smaller contact angle corresponds to higher wettability, which implies a more hydrophilic surface and results in better dispersibility in the medium. Water has been regularly used as the medium in contact angle determination [53] and was used in this contribution. Higher wettability using water as a medium denotes lower surface tension or higher free energy of the powder; therefore, it is expected that the powder dissolves easily in water [46]. HCT presented a higher contact angle than OLM, indicating higher wettability of OLM. The combination of drugs produced a decrease in contact angle values compared to the values observed in the individual drugs. The formulation containing OLM as a majority component exhibited a contact angle value quite similar to pure OLM. As wettability is closely related to solubility, the results suggest that the association of OLM and HCT would improve the water solubility of each drug. However, further analyses need to be done to correlate the contact angles and solubilities of this system.

### 3.5. Dissolution Test of HCT-OLM Tablets

The dissolution test aims to evaluate the adequate performance of a drug in a pharmaceutical product as well as the quality and reproducibility of the product from batch to batch. The assay was performed according to an in-house method that has been developed and validated accordingly with Costa Rica’s regulatory requirements as the OLM-HCT is not an official pharmacopeial product. Therefore, the dissolution medium used for these products was HCl 0.1 N in which both drugs were completely soluble.

The results obtained for the final F_1_ and F_2_ products are presented in Table 2. The appropriate performance of a dosage form administered orally is confirmed when the compliance with the dissolution requirements indicated in the specific USP monograph are fulfilled or by the pre-established criteria of the in-house developed methods [54]. In this scenario, OLM and HCT in both formulations, F_1_ and F_2,_ exhibited dissolution accordingly with the product specification in the evaluated time.

### 3.6. Solid-State and Physical Stability

The long-term stability of both formulations, F_1_ and F_2_, were evaluated through PXRD, DSC, FT-IR, and color measurements after 24 months of storage. 

**Figure 6:**
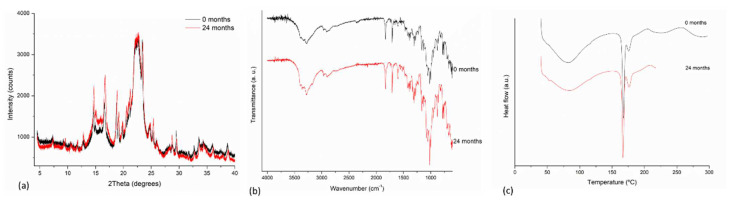
Solid-state stability determinations of F_1_ freshly prepared and after 24 months of storage of product (**a**) PXRD, (**b**) FT-IR, and (**c**) DSC curves.

Figure 6 shows the PXRD patterns, DSC curves, and FT-IR spectra comparing the freshly prepared product with the product after 24 months for F_1_ formulation. There are no differences observed between 0 months and 24 months products. Similar results were observed for the F_2_ product (See Figure S4). Further, the dissolution test results presented in Table 3 demonstrated an unaltered product after time of storage.

Finally, Table 4 presents the results obtained in the measurements of color. It shows that all the tablets, at 0 months and 24 months storage, have similar color because the CIELab coordinates are close. Additionally, the MCDM and ∆E00 parameters are very low. This means that there is no significant color variability or color change in the tables over time.

These results show the mean of tablet color in CIELab coordinates and the color dispersion and color change of OLM–HCT 20 mg (F_1_) and 40 mg (F_2_) from 0 months and 24 months. It was determined that in the case of F_1_, the Lightness (L*) remained in very similar ranges; the Chroma (C*) had a variation of one CIELab unit, while the angle remained approximately the same, so that the MCDM (Mean Color Differences from the Mean), which measures the variability in samples, was relatively stable both at 0 months and 24 months, although a decrease is probably due to the minimal loss of surface characteristics, which allows for greater confidence in the data obtained. In this case, the chromatic differential measured with the CIEDE2000 formula (∆E00) gives a difference of 1.32 units, which is barely noticeable, but could be due to surface discoloration caused by the aging of the tablets, which is necessary to monitor with sensitive equipment such as the DigiEye^®^. The results for F_2_ are similar, but show more variation in the Lightness (L*) than in the Chroma (C*); although the MCDM decreases with time, there is enough proportional similarity to the previous case. The chromatic differential (∆E00) is even smaller than F_1_.

## 4. Conclusions

Safe, effective, and stable FDC dosage forms containing OLM and HCT have been developed supported by a complete interaction and compatibility study. The FDC containing these drugs has been demonstrated to be the most effective therapy in significant BP and achieving BP control in many patients, and it is a well-tolerated therapy as well. The incidence of people with this medical condition uncontrolled is a concern in low- and middle-income countries. Therefore, it is important to develop reliable and accessible products. Studying drug–drug and drug–excipient interactions is an approach used to guarantee quality products by detecting chemical and/or physical instability during pharmaceutical product development. To achieve this goal, the evaluation of solid-state and physical properties through several techniques becomes essential. In this contribution, weak drug–drug interactions were observed to produce a eutectic mixture of OLM and HCT confirmed by DSC, PXRD, and FT-IR techniques. However, these interactions were not strong enough to provoke a phase transition or the formation of a new crystalline phase. In addition, drug–excipient interactions occurred between MC and both drugs, which were associated with an increase in the thermal stability of OLM and a slight decrease in the HCT one. In fact, these interactions did not represent incompatibilities because the analyses demonstrated that there was no influence on them in the final product performance. Further, solid-state characterization techniques applied to the long-term stability study indicated no changes in the solid-state and physical properties of the ageing product compared to the recently prepared, including the dissolution test. In relation to the colorimetric analysis, there are few perceptible variations in the color of the surface; it was a minimal change in the chromatic variables along time. Furthermore, the higher concentration of F_2_ exhibited less change on the color of the tablets surface.

## Figures and Tables

**Table 1:** Contact angle results.

Sample	Contact Angle °
OLM	41.4 ± 0.3
HCT	52.7 ± 0.3
OLM 20 mg-HCT 12.5 mg	26.1 ± 0.3
OLM 40 mg-HCT 12.5 mg	38.2 ± 0.3

**Table 2:** Drug content and percentage of labelled drug dissolved in the dissolution test for F_1_ and F_2_ formulations.

	F_1_		F_2_	
	OLM	HCT	OLM	HCT
Drug per tablet (mg)	38.8 ± 0.6	12.3 ± 0.3	19.6 ± 0.2	12.4 ± 0.4
Drug dissolved (%)	97.0	98.0	97.8	99.1

**Table 3:** Drug content and percentage of labelled drug dissolved in the dissolution test for F_1_ and F_2_ formulations after 24 months of storage.

	F_1_		F_2_	
	OLM	HCT	OLM	HCT
Drug per tablet (mg)	40.8 ± 0.2	13.0 ± 0.1	19.1 ± 0.3	12.6 ± 0.3
Drug dissolved (%)	101.0	104.0	96.0	106.0

**Table 4:** Results of color stability (MCDM, ∆E00) at 0 and 24 months for two different formulations, F_1_ and F_2._

Formulation	0 Months			24 Months			Color Stability Results	
	L*	C*	h°	L*	C*	h°	MCDM	∆E00
F_1_	95.65	3.71	105.17	93.91	2.79	100.99	0.43/0.39	1.32
F_2_	75.83	26.88	27.40	74.88	26.51	27.98	0.44/0.35	0.74

Measurements based on the color coordinates: Lightness (L*), Chroma (C*), and hue angle (h°).

## Data Availability

The data presented in this study are available upon request from the corresponding author.

## Supplementary Materials

The following are available online at https://www.mdpi.com/article/10.3390/pharmaceutics14020424/s1, Figure S1: Experimental PXRD patterns of each sampling of F_2_: (a) OLM and sample 1 (i.e., OLM+MC), (b) HCT and sample 2 (i.e., HCT+MC), (c) OLM, HCT and sampling 3 (i.e. sampling 1 + sampling 2), (d) Sampling 3 and samples from 4–7, Figure S2: FT-IR spectra of each sampling of F_2_: (a) OLM and sampling 1 (i.e., OLM+MC), (b) HCT and sampling 2 (i.e., HCT+MC), (c) OLM, HCT and sampling 3 (i.e. sampling 1 + 2), (d) Sampling 3 and samplings from 4–7, Figure S3: DSC curves of F_2_ (a) pure OLM, HCT and, sampling from 1 to 5, (b) sampling from 5 to 7 and mannitol, Figure S4: Solid-state stability determinations of F_2_ freshly prepared and after 24 months of storage product (a) PXRD, (b) FT-IR and (c) DSC curves.

## References

[1] World Health Organization A Global Brief on Hypertension. http://www.who.int/cardiovascular_diseases/publications/global_brief_hypertension/en/.

[2] World Health Organization Hypertension. https://www.who.int/health-topics/hypertension#tab=tab_1.

[3] Greathouse M. (2006). Olmesartan medoxomil combined with hydrochlorothiazide for the treatment of hypertension. Vasc. Health Risk Manag..

[4] Sarzani R., Giulietti F., Filipponi A., Marziali S., Ristori L., Buscarini S., Garbuglia C., Biondini S., Allevi M., Spannella F. (2021). The Number of Pills, Rather Than the Type of Renin—Angiotensin System Inhibitor, Predicts Ambulatory Blood Pressure Control in Essential Hypertensives on Triple Therapy: A Real-Life Cross-Sectional Study. Adv. Ther..

[5] Filipova E., Dineva S., Uzunova K., Pavlova V., Kalinov K., Vekov T. (2020). Combining angiotensin receptor blockers with chlorthalidone or hydrochlorothiazide—which is the better alternative? A meta-analysis. Syst. Rev..

[6] Kim D.-W., Weon K.Y. (2021). Pharmaceutical application and development of fixed-dose combination: Dosage form review. J. Pharm. Investig..

[7] Kjeldsen S.E., Os I., Høieggea A., Beckey K., Gleim G.W., Oparil S. (2005). Fixed-Dose Combinations in the Management of Hypertension. Am. J. Cardiovasc. Drugs.

[8] Chrysant S. (2004). Evaluation of antihypertensive therapy with the combination of olmesartan medoxomil and hydrochlorothiazide. Am. J. Hypertens..

[9] Sellin L., Stegbauer J., Laeis P., Rump L.C. (2005). Adding hydrochlorothiazide to olmesartan dose dependently improves 24-h blood pressure and response rates in mild-to-moderate hypertension. J. Hypertens..

[10] Lacourcière Y., Neutel J.M., Schumacher H. (2005). Comparison of fixed-dose combinations of telmisartan/hydrochlorothiazide 40/12.5 mg and 80/12.5 mg and a fixed-dose combination of losartan/hydrochlorothiazide 50/12.5 mg in mild to moderate essential hypertension: Pooled analysis of two multicenter, prospective, randomized, open-label, blinded-end point (PROBE) trials. Clin. Ther..

[11] Neutel J.M., Saunders E., Bakris G.L., Cushman W.C., Ferdinand K.C., Ofili E.O., Sowers J.R., Weber M.A. (2005). The Efficacy and Safety of Low- and High- Dose Fixed Combinations of Irbesartan/Hydrochlorothiazide in Patients With Uncontrolled Systolic Blood Pressure on Monotherapy: The INCLUSIVE Trial. J. Clin. Hypertens..

[12] Ball K.J., Williams P.A., Stumpe K.O. (2001). Relative efficacy of an angiotensin II antagonist compared with other antihypertensive agents. Olmesartan medoxomil versus antihypertensives. J. Hypertens..

[13] Quan A., Chavanu K., Merkel J. (2006). A Review of the Efficacy of Fixed-Dose Combinations Olmesartan Medoxomil/Hydrochlorothiazide and Amlodipine Besylate/Benazepril in Factorial Design Studies. Am. J. Cardiovasc. Drugs.

[14] Del Pinto R., Desideri G., Ferri C., Agabiti Rosei E. (2021). Real-world Antihypertensive Treatment Patterns, Treatment Adherence, and Blood Pressure Control in the Elderly: An Italian Awareness-raising Campaign on Hypertension by Senior Italia FederAnziani, the Italian Society of Hypertension and the Italian Federa. High Blood Press. Cardiovasc. Prev..

[15] Jeličić M.-L., Brusač E., Kurajica S., Cvetnić M., Amidžić Klarić D., Nigović B., Mornar A. (2021). Drug–Drug Compatibility Evaluation of Sulfasalazine and Folic Acid for Fixed-Dose Combination Development Using Various Analytical Tools. Pharmaceutics.

[16] Narang A.S., Desai D., Badawy S. (2012). Impact of Excipient Interactions on Solid Dosage Form Stability. Pharm. Res..

[17] Byrn S., Pfeiffer R., Stowell J. (1999). Solid-State Chemistry of Drugs.

[18] Shi Q., Chen H., Wang Y., Xu J., Liu Z., Zhang C. (2022). Recent advances in drug polymorphs: Aspects of pharmaceutical properties and selective crystallization. Int. J. Pharm..

[19] Bharate S.S., Bharate S.B., Bajaj A.N. (2010). Interactions and incompatibilities of pharmaceutical excipients with active pharmaceutical ingredients: A comprehensive review. J. Excip. Food Chem..

[20] ICH Harmonised Tripartite Guideline (2018). Q1F Stability Data Package for Registration Applications in Climatic Zones III and IV. ICH Harmon. Tripart. Guidel..

[21] Subert J., Cizmárik J. (2008). Application of instrumental colour measurement in development and quality control of drugs and pharmaceutical excipients. Pharmazie.

[22] International Commission on Illumination. https://cie.co.at/publications/international-standards.

[23] Murillo M.A., Rodríguez-Pulido F.J., Heredia F.J., Melgosa M., Pacheco J., Vargas R., Montero E., Gutiérrez D. (2019). Color evolution during a coating process of pharmaceutical tablet cores by random spraying. Color Res. Appl..

[24] Luo M.R., Cui G., Rigg B. (2001). The development of the CIE 2000 colour-difference formula: CIEDE2000. Color Res. Appl..

[25] Yu L. (1998). Physical characterization of polymorphic drugs: An integrated characterization strategy. Pharm. Sci. Technolo. Today.

[26] FDA Guidance for Industry ANDAs: Pharmaceutical Solid Polymorphism. https://www.fda.gov/regulatory-information/search-fda-guidance-documents/andaspharmaceutical-solid-polymorphism-chemistry-manufacturing-and-controls-information.

[27] Araya-Sibaja A.M., Fandaruff C., Wilhelm K., Vega-Baudrit J.R., Guillén-Girón T., Navarro-Hoyos M. (2020). Crystal Engineering to Design of Solids: From Single to Multicomponent Organic Materials. Mini. Rev. Org. Chem..

[28] Kapoor A.K., Mehta H.S., Nath A., Prasad M. (2011). Polymorphic forms of Olmesartan Medoxomil. International Patent.

[29] Qi M.-H., Chen W.-S., Zhou H., Chen J.-Y., Ren G.-B. (2017). Solution-mediated polymorphic transformation of amorphous form to Form I of olmesartan medoxomil in methanol-water mixture solvents. Cryst. Res. Technol..

[30] Saini A., Chadha R., Gupta A., Singh P., Bhandari S., Khullar S., Mandal S., Jain D.S. (2016). New conformational polymorph of hydrochlorothiazide with improved solubility. Pharm. Dev. Technol..

[31] Huang S., Williams R.O. (2018). Effects of the Preparation Process on the Properties of Amorphous Solid Dispersions. AAPS PharmSciTech.

[32] Einfalt T., Planinšek O., Hrovat K. (2013). Methods of amorphization and investigation of the amorphous state. Acta Pharm..

[33] Maréchal Y. (2007). The hydrogen bond: Formation, thermodynamic properties, classification. The Hydrogen Bond and the Water Molecule.

[34] Larkin P. (2011). Environmental dependence of vibrational spectra. Infrared and Raman Spectroscopy.

[35] Abdelquader M.M., Essa E.A., El Maghraby G.M. (2019). Inhibition of Co-Crystallization of Olmesartan Medoxomil and Hydrochlorothiazide for Enhanced Dissolution Rate in Their Fixed Dose Combination. AAPS PharmSciTech.

[36] Larkin P. (2011). General outline and strategies for IR and raman spectral Interpretation. Infrared and Raman Spectroscopy.

[37] Setu M.N.I., Mia M.Y., Lubna N.J., Chowdhury A.A. (2015). Preparation of Microcrystalline Cellulose from Cotton and its Evaluation as Direct Compressible Excipient in the Formulation of Naproxen Tablets. Dhaka Univ. J. Pharm. Sci..

[38] Pachuau L., Vanlalfakawma D.C., Tripathi S.K., Lalhlenmawia H. (2014). Muli bamboo (Melocanna baccifera) as a new source of microcrystalline cellulose. J. Appl. Pharm. Sci..

[39] Detoisien T., Arnoux M., Taulelle P., Colson D., Klein J.P., Veesler S. (2011). Thermal analysis: A further step in characterizing solid forms obtained by screening crystallization of an API. Int. J. Pharm..

[40] Reading M., Craig D.Q.M. (2007). Principles of differential scanning calorimetry. Thermal Analysis of Pharmaceutical Compounds.

[41] Galwey A.K., Craig D.Q.M. (2007). Thermogravimetric analysis: Basic principles. Thermal Analysis of Pharmaceutical Compounds.

[42] Jain H., Khomane K.S., Bansal A.K. (2014). Implication of microstructure on the mechanical behaviour of an aspirin–paracetamol eutectic mixture. CrystEngComm.

[43] Tran T.T.D., Tran P.H.L. (2020). Molecular Interactions in Solid Dispersions of Poorly Water-Soluble Drugs. Pharmaceutics.

[44] Cherukuvada S., Nangia A. (2014). Eutectics as improved pharmaceutical materials: Design, properties and characterization. Chem. Commun..

[45] Vippagunta S.R., Wang Z., Hornung S., Krill S.L. (2007). Factors Affecting the Formation of Eutectic Solid Dispersions and Their Dissolution Behavior. J. Pharm. Sci..

[46] Hyun S.-M., Lee B.J., Abuzar S.M., Lee S., Joo Y., Hong S.-H., Kang H., Kwon K.-A., Velaga S., Hwang S.-J. (2019). Preparation, characterization, and evaluation of celecoxib eutectic mixtures with adipic acid/saccharin for improvement of wettability and dissolution rate. Int. J. Pharm..

[47] Sathisaran I., Dalvi S. (2018). Engineering Cocrystals of Poorly Water-Soluble Drugs to Enhance Dissolution in Aqueous Medium. Pharmaceutics.

[48] Vasconcelos T., Sarmento B., Costa P. (2007). Solid dispersions as strategy to improve oral bioavailability of poor water soluble drugs. Drug Discov. Today.

[49] International Conference on Harmonisation ICH Harmonised Tripartite Guideline Q6A Test Procedures and Acceptance Criteria for New Drug Substance. https://www.ich.org/fileadmin/Public_Web_Site/ICH_Products/Guidelines/Quality/Q6A/Step4/Q6Astep4.pdf.

[50] Pilpel N., Britten J.R., Onyekweli A.O., Esezobo S. (1991). Compression and Tableting of Pharmaceutical Powders at Elevated Temperatures. Int. J. Pharm..

[51] Bi M., Hwang S.-J., Morris K.R. (2003). Mechanism of eutectic formation upon compaction and its effects on tablet properties. Thermochim. Acta.

[52] Park S.-J., Seo M.-K. (2011). Solid-liquid interface. Interface Science and Composites.

[53] Lu Y., Tang N., Lian R., Qi J., Wu W. (2014). Understanding the relationship between wettability and dissolution of solid dispersion. Int. J. Pharm..

[54] FDA Guidance for Industry ANDAs: Analytical Procedures and Methods Validation for Drugs and Biologics. https://www.fda.gov/files/drugs/published/Analytical-Procedures-and-Methods-Validation-for-Drugs-and-Biologics.pdf.

