# Supplementary material for: Interaction and Compatibility Studies in the Development of Olmesartan Medoxomil and Hydrochlorothiazide Formulations under a Real Manufacturing Process
Source: Pharmaceutics. 2022 Feb 16;14(2):424. doi: 10.3390/pharmaceutics14020424 (PMC8875139; doi:10.3390/pharmaceutics14020424)
Supplement: Supplementary file 1 [file pharmaceutics-14-00424-s001.zip › pharmaceutics-1504923-supplementary.pdf]

pharmaceutics

Supplementary Materials: Interaction and Compatibility Studies in the Development of Olmesartan Medoxomil and Hydrochlorothiazide Formulations under a Real Manufacturing Process

Mac Arturo Murillo-Fernández, Ernesto Montero-Zeledón, Ariadna Abdala-Saiz, José Roberto Vega-Baudrit and Andrea Mariela Araya-Sibaja

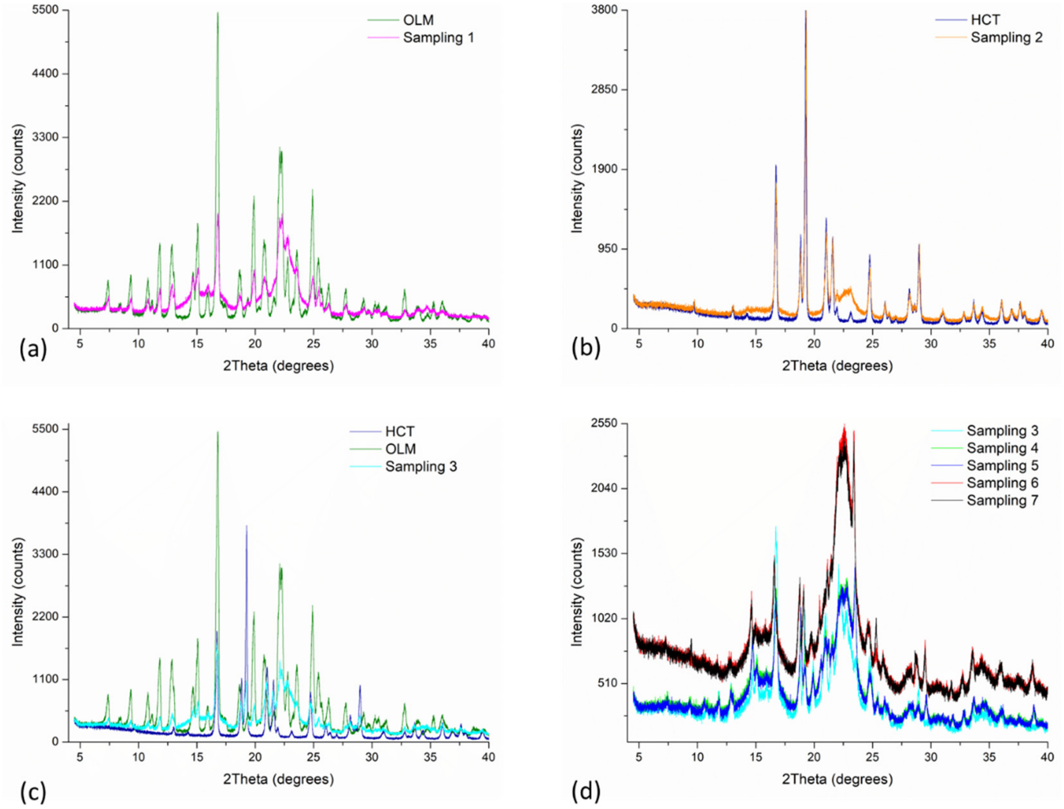

Figure S1. Experimental PXRD patterns of each sampling of F_2: (a) OLM and sample 1 (i.e., OLM+MC), (b) HCT and sample 2 (i.e., HCT+MC), (c) OLM, HCT and sampling 3 (i.e. sampling 1 + sampling 2), (d) Sampling 3 and samples from 4-7.

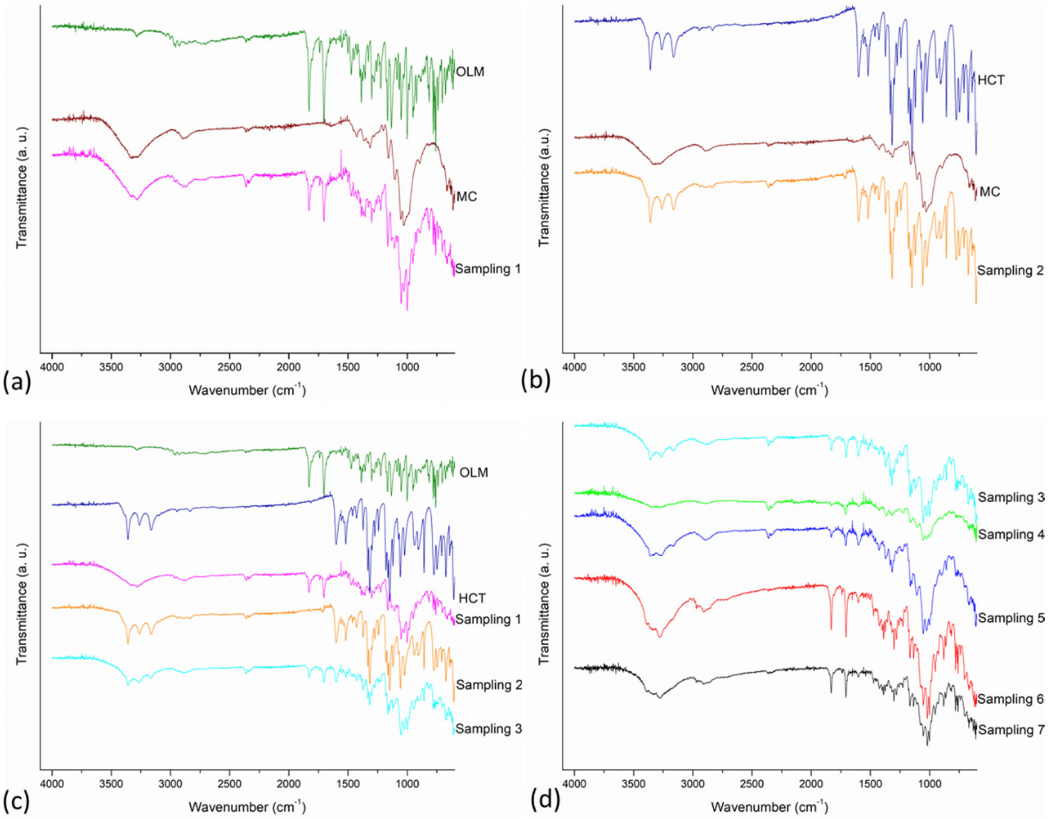

Figure S2. FT-IR spectra of each sampling of F_2: (a) OLM and sampling 1 (i. e., OLM+MC), (b) HCT and sampling 2 (i.e., HCT+MC), (c) OLM, HCT and sampling 3 (i.e. sampling 1 + 2), (d) Sampling 3 and samplings from 4-7.

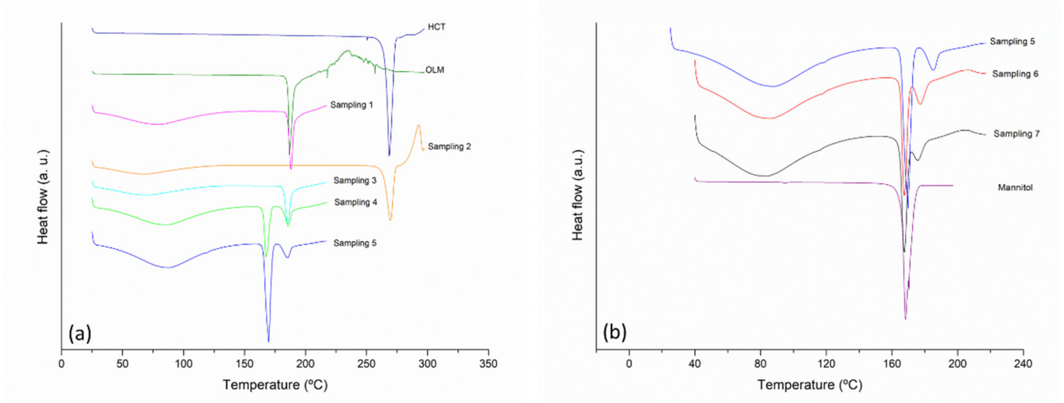

Figure S3. DSC curves of F_2 (a) pure OLM, HCT and, sampling from 1 to 5, (b) sampling from 5 to 7 and mannitol.

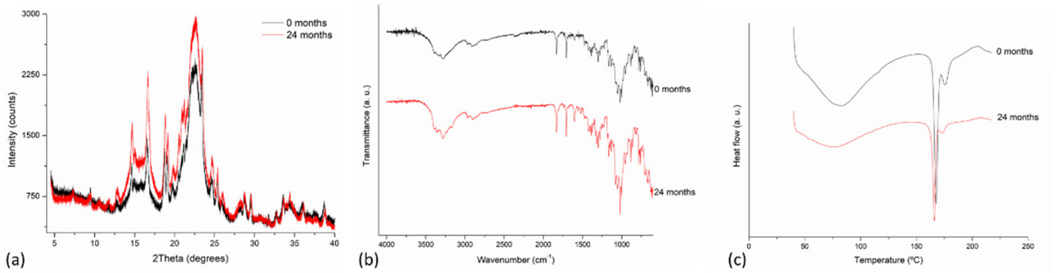

Figure S4. Solid-state stability determinations of F_2 freshly prepared and after 24 months of storage product (a) PXRD, (b) FT-IR and (c) DSC curves.
